# Genomic, Biochemical, and Modeling Analyses of Asparagine Synthetases from Wheat

**DOI:** 10.3389/fpls.2017.02237

**Published:** 2018-01-15

**Authors:** Hongwei Xu, Tanya Y. Curtis, Stephen J. Powers, Sarah Raffan, Runhong Gao, Jianhua Huang, Monika Heiner, David R. Gilbert, Nigel G. Halford

**Affiliations:** ^1^Biotechnology Research Institute, Shanghai Academy of Agricultural Sciences, Shanghai, China; ^2^Department of Plant Sciences, Rothamsted Research, Harpenden, United Kingdom; ^3^Department of Computational and Analytical Sciences, Rothamsted Research, Harpenden, United Kingdom; ^4^Department of Computer Science, Brandenburg University of Technology Cottbus-Senftenberg, Cottbus, Germany; ^5^Department of Computer Science, College of Engineering, Design and Physical Sciences, Brunel University London, Uxbridge, United Kingdom

**Keywords:** wheat, asparagine synthetase, acrylamide, food safety, enzyme activity, mathematical modeling

## Abstract

Asparagine synthetase activity in cereals has become an important issue with the discovery that free asparagine concentration determines the potential for formation of acrylamide, a probably carcinogenic processing contaminant, in baked cereal products. Asparagine synthetase catalyses the ATP-dependent transfer of the amino group of glutamine to a molecule of aspartate to generate glutamate and asparagine. Here, asparagine synthetase-encoding polymerase chain reaction (PCR) products were amplified from wheat (*Triticum aestivum*) cv. Spark cDNA. The encoded proteins were assigned the names TaASN1, TaASN2, and TaASN3 on the basis of comparisons with other wheat and cereal asparagine synthetases. Although very similar to each other they differed slightly in size, with molecular masses of 65.49, 65.06, and 66.24 kDa, respectively. Chromosomal positions and scaffold references were established for *TaASN1, TaASN2*, and *TaASN3*, and a fourth, more recently identified gene, *TaASN4*. *TaASN1, TaASN2*, and *TaASN4* were all found to be single copy genes, located on chromosomes 5, 3, and 4, respectively, of each genome (A, B, and D), although variety Chinese Spring lacked a *TaASN2* gene in the B genome. Two copies of *TaASN3* were found on chromosome 1 of each genome, and these were given the names *TaASN3.1* and *TaASN3.2*. The TaASN1, TaASN2, and TaASN3 PCR products were heterologously expressed in *Escherichia coli* (*TaASN4* was not investigated in this part of the study). Western blot analysis identified two monoclonal antibodies that recognized the three proteins, but did not distinguish between them, despite being raised to epitopes SKKPRMIEVAAP and GGSNKPGVMNTV in the variable C-terminal regions of the proteins. The heterologously expressed TaASN1 and TaASN2 proteins were found to be active asparagine synthetases, producing asparagine and glutamate from glutamine and aspartate. The asparagine synthetase reaction was modeled using SNOOPY^®^ software and information from the BRENDA database to generate differential equations to describe the reaction stages, based on mass action kinetics. Experimental data from the reactions catalyzed by TaASN1 and TaASN2 were entered into the model using Copasi, enabling values to be determined for kinetic parameters. Both the reaction data and the modeling showed that the enzymes continued to produce glutamate even when the synthesis of asparagine had ceased due to a lack of aspartate.

## Introduction

Asparagine is an important nitrogen storage and transport molecule in many plant species due to its relatively high nitrogen to carbon ratio (2:4, compared with 2:5 for glutamine, 1:5 for glutamic acid and 1:4 for aspartic acid, for example) and its relative chemical inertia (Lea et al., 2007). It accumulates in its free (non-protein) form in response to a range of abiotic and biotic stresses, as well as during normal physiological processes such as seed germination (Lea et al., 2007). In wheat grain it accumulates to very high levels in response to sulfur deficiency (Muttucumaru et al., 2006; Granvogl et al., 2007; Curtis et al., 2009, 2018) and poor disease control (Curtis et al., 2016). There are also large differences in the free asparagine concentration of grain from different wheat varieties (Curtis et al., 2018). Understanding the mechanisms that control free asparagine accumulation is important for improving crop yield and stress resistance. However, more pressingly, it also has implications for food safety because free asparagine is a precursor for acrylamide formation (reviewed by Halford et al., 2012; Curtis et al., 2014).

Acrylamide is a processing contaminant that forms during high-temperature cooking and processing, particularly as a result of frying, roasting, and baking. It is classed as a probable (Group 2a) carcinogen by the International Agency for Research on Cancer (1994) and has reproductive and neurotoxicological effects at high doses (Friedman, 2003). The European Food Safety Authority (EFSA) Expert Panel on Contaminants in the Food Chain (CONTAM) stated in its 2015 report that the margins of exposure for acrylamide indicate a concern for neoplastic effects (EFSA Panel on Contaminants in the Food Chain [Contam], 2015). The European Commission issued “Indicative Values” for the presence of acrylamide in food in 2011, based on results reported to EFSA (European Food Safety Authority, 2011), and reduced them for many product types in 2013 (European Commission, 2013). If a product is found to exceed the Indicative Value, the relevant food safety authority should take action to ensure that the manufacturer addresses the problem. Furthermore, the European Commission has just approved strengthened risk management measures including compulsory Codes of Practice and the renaming of Indicative Values as Benchmark Levels, with reduced Benchmark Levels for many products (European Commission, 2017). The proposals also include a specific reference to the setting of mandatory Maximum Levels for acrylamide in certain foods, stating that this should be considered following the adoption of the new regulations. The proposals will come before the European Parliament and European Council in 2017 and could be in force by early 2018.

The predominant route for the formation of acrylamide is via a Strecker-type degradation of free asparagine by highly reactive carbonyl compounds produced within the Maillard reaction (Mottram et al., 2002; Stadler et al., 2002; Zyzak et al., 2003), and free asparagine concentration is the main determinant of acrylamide-forming potential in cereal grains (Muttucumaru et al., 2006; Granvogl et al., 2007; Curtis et al., 2009, 2010; Postles et al., 2013). This has re-invigorated interest in the enzymes involved in asparagine synthesis and breakdown, and other metabolic pathways that could impact on free asparagine concentrations.

Asparagine synthesis is catalyzed by the enzyme asparagine synthetase, and occurs by the ATP-dependent transfer of the amino group of glutamine to a molecule of aspartate to generate glutamate and asparagine. Two asparagine synthetase genes were cloned from wheat (*Triticum aestivum*) by Wang et al. (2005) and called *TaASN1* and *TaASN2*. *TaASN1* expression in seedlings was shown to be up-regulated by treatment with abscisic acid, and by salt and osmotic stress (Wang et al., 2005). Subsequently, its expression in leaves was shown to be induced by sulfur deficiency, but to be greatly reduced when a general control non-derepressible-2-type protein kinase, TaGCN2, was over-expressed (Byrne et al., 2012). In 2016, two additional genes, *TaASN3* and *TaASN4*, were identified (Gao et al., 2016), although *TaASN4* was only discovered from wheat genome data and has not yet been cloned or characterized. The expression of *TaASN1–3* was studied in different tissues and in response to nutrition (Gao et al., 2016). Notably, the expression of *TaASN2* in the embryo and endosperm during mid to late grain development was shown to be the highest of any of the genes in any tissue, although *TaASN1* was most responsive to sulfur supply.

Maize (*Zea mays*) and barley (*Hordeum vulgare*) also have four differentially-expressed asparagine synthetase genes (Todd et al., 2008; Avila-Ospina et al., 2015), suggesting that this is typical of the cereals. However, a full picture of the role of the different asparagine synthetases will only emerge when the kinetic parameters of the enzymes have been measured. This is problematic because asparagine synthetase activity in plant tissues is difficult to purify and measure (Joy et al., 1983; Snapp and Vance, 1986; Kudiyarova et al., 2013), probably because of the presence of asparaginase activities and natural inhibitors (Rognes, 1980). There has also been a scarcity of antibodies for immunological analysis of purified or expressed proteins. However, the enzymes encoded by three of the maize genes have been analyzed after heterologous expression in *Escherichia coli* and been shown to have significant differences in kinetic properties (Duff et al., 2011). The aim of this study was to characterize the wheat asparagine synthetase gene family and to compare the enzymes encoded by *TaASN1* and *TaASN2*, the two genes that are most highly expressed in the grain.

## Materials and Methods

### Plant Materials and Growth Conditions

Wheat (*T. aestivum*) cv. Spark seeds were surface-sterilized as described by Gao et al. (2016), and germinated in a growth room in small containers. After 7 days, seedlings were harvested, flash frozen in liquid nitrogen, and then stored at -80°C ready for use.

### Molecular Cloning of *TaASN1, TaASN2*, and *TaASN3*

RNA was extracted using the hot phenol method (Verwoerd et al., 1989), with some modification, as described previously (Postles et al., 2016). It was used as a template for first-strand cDNA synthesis using SuperScript III^®^ first-strand synthesis supermix (Invitrogen, supplied by Thermo Fisher Scientific, Hemel Hempstead, United Kingdom). The full-length coding sequences of *TaASN1, TaASN2*, and *TaASN3* were then amplified by polymerase chain reaction (PCR). “Forward” and “reverse” primers for *TaASN1* were 5′-ccggaattcATGTGCGGCATACTGGC and 5′-ccgctcgagAACTCTCAATTGCGACACCAG (lower case letters denote additional nucleotides that were added to incorporate *Eco*RI and *Xho*I restriction sites at either end of the PCR product). “Forward” and “reverse” primers for *TaASN2* were 5′-ccggaattcATGTGCGGCATACTAGCGGTG and 5′-ccgctcgagAAGTCTCAATGGCAAC, while for *TaASN3* they were 5′-ccggaattcATGTGCGGCATCCTCGC and 5′-ataagaatgcggccgcAAACAGCAGCTGCTGGAACA. The additional nucleotides on the “reverse” primer for *TaASN3* incorporated a *Not*I restriction site.

All products were amplified using Phusion^®^ High-Fidelity DNA polymerase (New England Biolabs, Hitchin, United Kingdom). The cycling conditions were: 30 s denaturation at 98°C, followed by 35 cycles of 10 s at 98°C, 30 s at 63°C, and 30 s at 72°C, with a final extension period at 72°C for 10 min. The resulting PCR products were purified using the Wizard PCR Clean-up system (Promega, Southampton, United Kingdom) and ligated into the pGEM-T Easy Vector (Promega, Southampton, United Kingdom) using the restriction sites incorporated during the PCR. Nucleotide sequence analysis was performed by MWG Biotech (Wolverhampton, United Kingdom) and contigs were assembled using ContigExpress or Geneious Version 8^1^ (Kearse et al., 2012). Amino acid sequence alignments were also performed using Geneious Version 8.

### Genomic Analysis

DeCypher Tera-BLASTN Search Nucleic Query vs. Nucleic Database was used to assess the wheat asparagine synthetase gene sequences. The NR_Gene_v0.4 scaffold wheat genome was used as the database of choice (PLANT_T.aestivum_NRgene_v0.4_scaf), and the cDNA nucleotide sequences for *TaASN1* (GenBank BT009245), *TaASN2* (GenBank BT009049), and *TaASN3* (GenBank AK333183) were used as the query sequences.

The returned scaffolds were downloaded and aligned to the cDNAs using the Geneious Version 8 software package (pairwise alignment was run using the Geneious Alignment algorithm on its default settings; multiple alignments were run using the Consensus Align algorithm, again on its default settings). The aligned consensus sequences were then used to search the *T. aestivum* TGACv1 (Genomic sequence) database^2^ to assess chromosomal positioning. The returned genes from the TGAC database were then re-aligned to the original cDNA sequences to confirm gene identity.

*TaASN4* was identified through its divergence from the other wheat asparagine synthetase sequences. The TGAC sequence was confirmed through re-alignments to both the TGAC and NR-Gene databases. BLAST searches using the PLANT_T.aestivum_nt_w7984 database were used to further confirm gene identity.

### Heterologous Expression of *TaASN1, TaASN2*, and *TaASN3* in *E. coli*

The PCR products were excised from the PGEM^®^-T vector and ligated into the specialist expression vector, pET-30a (Novagen, United Kingdom) to produce plasmids pET-30a–TaASN1, pET-30a–TaASN2, and pET-30a–TaASN3. These were maintained in *E. coli* NovaBlue cells (Novagen, United Kingdom), which carry *recA* and *endA* mutations, and transferred to RosettaBlue^TM^ cells (Novagen, United Kingdom) for high levels of expression of the ASN1–3 proteins. Single colonies of the cells carrying the plasmids were inoculated into medium containing 15 μg/mL kanamycin and 34 μg/mL chloramphenicol. The bacteria were grown at 37°C with shaking until they had reached mid-log phase (OD 600 between 0.6 and 1.0). The culture was then split between two flasks, and isopropyl β-D-1-thiogalactopyranoside (IPTG) was added to one of the flasks to a final concentration of 1 mM in order to induce expression of the asparagine synthetase gene carried by the plasmid. The other flask acted as an “un-induced” control. The bacteria were incubated with shaking at 27°C for a further 3 h, then harvested by centrifugation and stored at -80°C until further use.

The use of the pET30a plasmid meant that the asparagine synthetase proteins were synthesized with a six-residue histidine N-terminal tag, and could therefore be extracted and purified using the nickel-nitrilotriacetic acid (Ni-NTA) purification system (Invitrogen, supplied by Thermo Fisher Scientific, Hemel Hempstead, United Kingdom). Bacterial cells were pelleted and lysed. Proteins in inclusion bodies were solubilized using NuPAGE^®^ LDS-sample buffer and NuPAGE^®^ Sample Reducing Agent and the proteins were separated on 4–12% Bis-Tris gels (Invitrogen, supplied by Thermo Fisher Scientific, Hemel Hempstead, United Kingdom). Protein concentration was assayed using a Bicinchoninic Acid Kit (Sigma–Aldrich, Gillingham, United Kingdom).

### Western Analysis

Monoclonal antibodies were produced by Abmart (Shanghai, China). Peptides were synthesized corresponding to probable epitopes in the C-terminal region of the TaASN1, TaASN2, and TaASN3 proteins where the amino acid sequences show less similarity with each other. Antibodies were raised to four different peptides for each of the three proteins. For western analysis, soluble proteins were separated by electrophoresis on NuPAGE^®^ Novex^®^ 4–12% Bis-Tris gels and transferred to polyvinylidene fluoride membranes (13 cm × 8 cm) using the iBlot^®^ Gel Transfer Device (Invitrogen, supplied by Thermo Fisher Scientific, Hemel Hempstead, United Kingdom). Immunodetection was performed with the antibody in a 1:1000 dilution for 2 h at room temperature, after which the membrane was incubated for 1 h at room temperature with 1:15,000 horseradish peroxidase-conjugated goat anti-mouse IgG (Invitrogen, supplied by Thermo Fisher Scientific, Hemel Hempstead, United Kingdom). Bands representing proteins that had reacted with the anti-asparagine synthetase antibody were visualized with ECL^TM^ Western Blotting Detection Reagents (GE Healthcare, Amersham, United Kingdom), and signals were quantified by scanning densitometry using Quantity One software (Bio-Rad Laboratories, Hemel Hempstead, United Kingdom).

### Asparagine Synthetase Activity Assay

Purified asparagine synthetase proteins, TaASN1 and TaASN2, were added to an assay buffer of 100 mM HEPES (pH 7.6), 1.6 mM aspartate, 10 mM glutamine, 10 mM ATP, 10 mM MgCl_2_, and 1 mM DTT and incubated at 30°C. Aliquots (100 μL) were removed after 1.5, 2.5, 3.5, 5, 15, 25, and 35 min, placed in a 96-well filter plate and mixed with 100 μL of 10% trichloroacetic acid to stop the reaction (Todd et al., 2008; Duff et al., 2011; Kudiyarova et al., 2013). Two replicate assays were done.

The free asparagine and glutamate produced in the reaction were detected after derivatization with *o*-phthalaldehyde reagent (Sigma–Aldrich, Gillingham, United Kingdom). The fluorescent derivative was measured by high performance liquid chromatography (HPLC) using a Waters Alliance 2795 HPLC system fitted with a Waters 474 Scanning Fluorescence Detector (Waters, Elstree, United Kingdom). A Symmetry C_18_, 4.6 mm × 150 mm column (for particle size 3.5–5 μm) (Waters, Elstree, United Kingdom) was used, and the fluorescence detector was set with an excitation wavelength of 340 nm and emission wavelength of 455 nm. For calibration, standards were used to provide areas under the HPLC peaks corresponding to asparagine and glutamate concentrations of 0, 5, 10, 15, and 20 nmol. The areas were modeled on the concentrations using linear regression, so that, by inverting the resulting linear equation, estimated concentrations of the amino acids and the asparagine synthetase enzyme could be made given HPLC areas for the sample aliquots taken at the seven sampling time points. Standards were run separately for each experiment.

### Modeling the Asparagine Synthetase Reaction

A model for the reactions catalyzed by asparagine synthetases TaASN1 and TaASN2 was constructed using the SNOOPY^®^ tool^3^ (Heiner et al., 2012) for designing, animating, and simulating Petri Nets. The model was then exported to Copasi 4.16 (Build 104; Hoops et al., 2006). Data for the reaction parameters were taken from the Brenda enzyme database^4^.

## Results

### Molecular Cloning and Identification of Three Asparagine Synthetase-Encoding cDNAs from Wheat (*T. aestivum*)

Gao et al. (2016) identified three distinct asparagine synthetase gene nucleotide sequences in the GenBank database: *TaASN1* (Wang et al., 2005; GenBank AY621539; BT009245), *TaASN2* (GenBank BT009049), and *TaASN3* (GenBank AK333183). *TaASN1* and *TaASN2* were already annotated as asparagine synthetases, but the *TaASN3* entry had not been up to that point. Gao et al. (2016) also identified a fourth gene, *TaASN4*, from a BLAST search of wheat genome data ^5^ (Wilkinson et al., 2012). *TaASN4* is present in cultivated and wild rice (*Oryza sativa* and *Oryza brachyantha*), *Brachypodium distachyon, Aegilops tauschii*, foxtail millet (*Setaria italica*), and maize (*Z. mays*), as well as wheat (Gao et al., 2016), but to date has not been cloned from or characterized in wheat.

Gao et al. (2016) described the differential expression of *TaASN1, TaASN2*, and *TaASN3* in different wheat tissues and in response to nitrogen and sulfur feeding, with expression of *TaASN2* in the embryo and to a lesser extent the endosperm of the grain during mid-development being far higher than the expression of any of the genes in any other tissue, although *TaASN1* showed most response to nutrition. This suggests that *TaASN2* expression in the grain is the primary determinant of asparagine levels, either for protein synthesis or accumulation in the free form, rather than import of free asparagine from other tissues, at least under normal (nutrient-sufficient) conditions, making *TaASN2* a potential target for genetic interventions to reduce free asparagine accumulation in wheat grain. However, modeling of the processes controlling the accumulation of free asparagine in order to confirm this requires information on the kinetic parameters of asparagine synthetase enzymes to add to the data on gene expression, and this was the aim of the current study.

To that end, *TaASN1, TaASN2*, and *TaASN3* PCR products were amplified from wheat cv. Spark using primers designed from published sequences (see section “Materials and Methods”). *TaASN1* from this variety was found to encode a protein of 585 amino acid residues with a molecular weight of 65.49 kDa, while *TaASN2* encoded a slightly smaller protein of 581 residues, molecular weight 65.06 kDa, and *TaASN3* a slightly larger protein of 591 residues, molecular weight 66.24 kDa. The nucleotide sequences have been deposited in the GenBank database and been given accession numbers KY937995, KY937996, and KY937997. Comparisons of the derived amino acid sequences of the proteins with those encoded by the nucleotide sequences already in the database confirmed that the three PCR products were derived from *TaASN1, TaASN2*, and *TaASN3* (**Table 1**).

**Table 1:** Amino acid sequence identity between the asparagine synthetases encoded by the PCR products amplified from wheat cv. Spark and wheat asparagine synthetases from the GenBank database.

	AY621539 (TaASN1)	BT009049 (TaASN2)	AK333183 (TaASN3)
Spark TaASN1	99%	88%	77%
Spark TaASN2	88%	100%	78%
Spark TaASN3	80%	78%	97%

**FIGURE 1:**
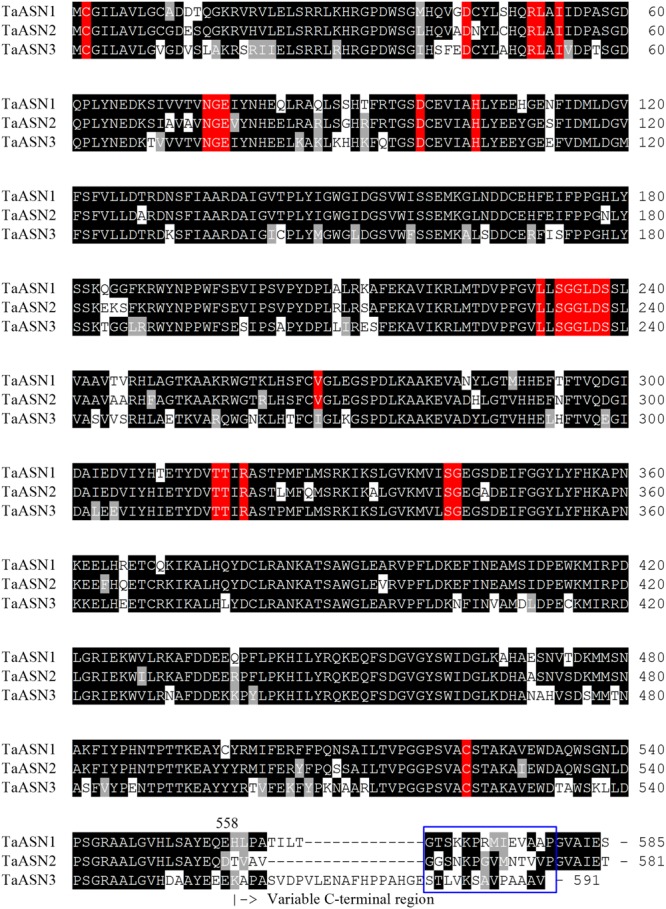
Amino acid sequence alignment of TaASN1, TaASN2, and TaASN3 proteins from wheat (*Triticum aestivum*) cv. Spark. Identical residues at the same position are highlighted in black, except for residues known to be critical for the function of the enzyme (see text), which are highlighted in red. Similar residues at the same position (conservative substitutions) are highlighted in gray. The region corresponding to peptides used to raise the two monoclonal antibodies that showed highest specificity for the asparagine synthetase proteins is indicated with a blue box.

The amino acid sequences of the three proteins are aligned in **Figure 1**. All three share conserved amino acid residues typical of asparagine synthetases (highlighted in red in **Figure 1**), including the essential residues of a *purF*-type glutamine-binding site, Cys^2^, Asp^34^, and His^104^ (Mei and Zalkin, 1989), and other residues important for glutamine binding and positioning that have been identified from the *E. coli* AsnB enzyme (Arg^50^, Leu^51^, Ile^53^, Asn^75^, Gly^76^, Glu^77^, and Asp^98^; Larsen et al., 1999). Residues Thr^316^, Thr^317^, Arg^319^, and Cys^523^ are involved in the binding of aspartate and ATP (Boehlein et al., 1994a,b, 1997a,b), while Leu^231^, Val^267^, Ser^341^, and Gly^342^ have been recognized as the anchoring points for the AMP moiety (Larsen et al., 1999). Interestingly, Val^267^ is replaced with a different hydrophobic residue, Ile, in TaASN3. Lastly, the Ser, Gly, Gly, Leu, Asp, Ser motif beginning at position 233 is conserved in all of the asparagine synthetases characterized to date and may be involved in pyrophosphate binding (Richards and Schuster, 1998).

### Gene Structure and Location

BLAST searches were performed of the wheat (*T. aestivum*) scaffold genome in the non-redundant genome database (NR_Gene_v0.4) using the cDNA sequences for *TaASN1, TaASN2*, and *TaASN3* (Gao et al., 2016). This search also identified scaffolds for *TaASN4*, which to date has not been cloned from bread wheat (Gao et al., 2016). The returned scaffolds were aligned to the cDNAs to identify exons and introns, and the results confirmed by searches of the *T. aestivum* TGACv1 genomic sequence. The consensus sequences derived from these alignments were then used to search the Ensembl wheat database to determine the chromosomal positioning of the genes. Note that both the NR and TGAC genome data are from variety Chinese Spring.

The chromosomal positions and scaffold references for the genes are given in **Table 2**. *TaASN1, TaASN2*, and *TaASN4* were all found to be single copy genes, located on chromosomes 5, 3, and 4, respectively, of each genome (A, B, and D), except that *TaASN2* was not present in the B genome. Analysis of unpublished wheat genome data (A. Huttly, Rothamsted Research, personal communication) suggests that not all wheat varieties lack a *TaASN2* gene on chromosome 3B, but the relative prevalence of the presence or absence of a B genome *TaASN2* gene cannot yet be assessed. In the case of *TaASN3*, there were two copies on chromosome 1 of each genome, and these were given the names *TaASN3.1* and *TaASN3.2*.

**Table 2:** Chromosomal position and scaffold references for wheat (*Triticum aestivum*) cv. Chinese Spring asparagine synthetase genes (*TaASN1–4*) (PLANT_*T. aestivum*_NRgene_v0.4_scaf).

Gene	Chromosomal position	NR_gene v0.4 scaffold reference	TGACv1 scaffold reference
*TaASN1*	5AL	10829_chr5A	376022_5AL:32188-35544
	5BL	86991_chr5B	404794_5BL:130599-132056
	5DL	24580_chr5D	438333_5DL:8628-10259
*TaASN2*	3AS	147930_chr3A	210989_3AS:73,515-78,055
	3DS	57063_chr3D	271746_3DS:53902-54460
*TaASN3*	1AL	72517_chr1A	004377_1AL:6267-12623
	1BL	94459_chr1B	032370_1BL:29616-29990
	1DL	40616_chr1D	061978_1DL:28081-28357
	1AL	81741_chr1A	002273_1AL:9143-9387
	1BL	95194_chr1B	031075_1BL:62894-63123
	1DL	86160_chr1D	061247_1DL:39750-39978
*TaASN4*	4AS	103865_chr4B	641929_U:112608-114682
	4BL	60431_chr4A	308427_4AS:31581-32567
	4DL	71289_chr4D	342578_4DL:59,248-59,851

**FIGURE 2:**
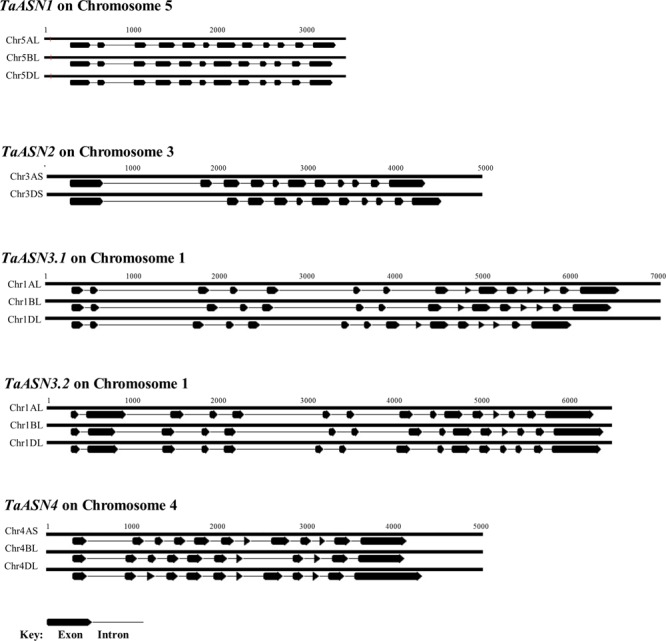
Diagrammatic representation of the gene structures of *TaASN1, TaASN2, TaASN3.1, TaASN3.2*, and *TaASN4*.

The structures of the genes are shown in **Figure 2**, illustrating the considerable divergence of intron/exon patterns between the different genes, but conservation of structure within each group of homeologs. The three *TaASN1* homeologs, on chromosomes 5A, B, and D, are the shortest at approximately 3 kb from the ATG translation start codon to the translation stop codon, including 12 exons. The two *TaASN2* homeologs are approximately 4 kb in length, with 11 exons, and the three *TaASN3.1* and *TaASN3.2* homeologs just over and just under 6 kb, respectively, making them the longest group. The two *TaASN3* genes share a similar intron/exon pattern, with 15 exons. The three *TaASN4* homeologs are just under 4 kb in length, with 12 exons, except that the *TaASN4* gene on chromosome 4B lacks exon 8. Clearly, this deletion may affect the activity of the enzyme encoded by the gene.

### Heterologous Expression and Purification of TaASN1, TaASN2, and TaASN3

The TaASN1, TaASN2, and TaASN3 PCR products were sub-cloned into vector pET-30a and the resulting plasmids transformed into *E. coli* Rosetta DE3 cells to enable expression of the asparagine synthetase proteins. Use of this system meant that the proteins were synthesized with a six-residue histidine “tag,” enabling them to be purified on a Ni-NTA agarose column. Expression of the proteins was induced by addition of IPTG to the cell culture medium.

**FIGURE 3:**
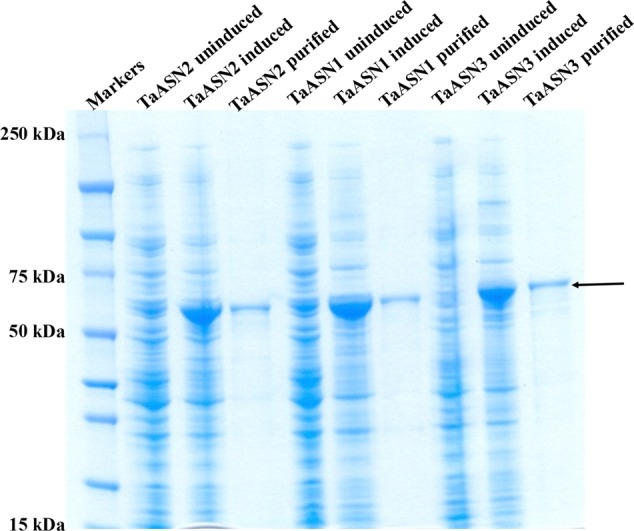
SDS-polyacrylamide gel electrophoresis of extracts of *E. coli* cells expressing wheat asparagine synthetases: TaASN1, TaASN2, and TaASN3. Expression of the proteins was induced by addition of IPTG to the bacterial cell cultures. An uninduced control is included for each protein, and each protein is also shown after purification on a nickel-nitrilotriacetic acid (Ni-NTA) agarose column. The arrow indicates the position of the expressed proteins.

The result of SDS-polyacrylamide gel electrophoresis (SDS-PAGE) of the expressed TaASN1, TaASN2, and TaASN3 proteins in both crude *E. coli* lysates and after purification on the column is shown in **Figure 3**. The proteins were solubilized by addition of NuPAGE^®^ LDS-sample buffer, which contains lithium dodecyl sulfate at a pH of 8.4, and NuPAGE^®^ Sample Reducing Agent, which contains dithiothreitol. An un-induced control is also shown (**Figure 3**) for each protein. The result showed the TaASN proteins to be highly expressed and readily purified.

### Western Analysis; Screening of Panel of Antibodies

**FIGURE 4:**
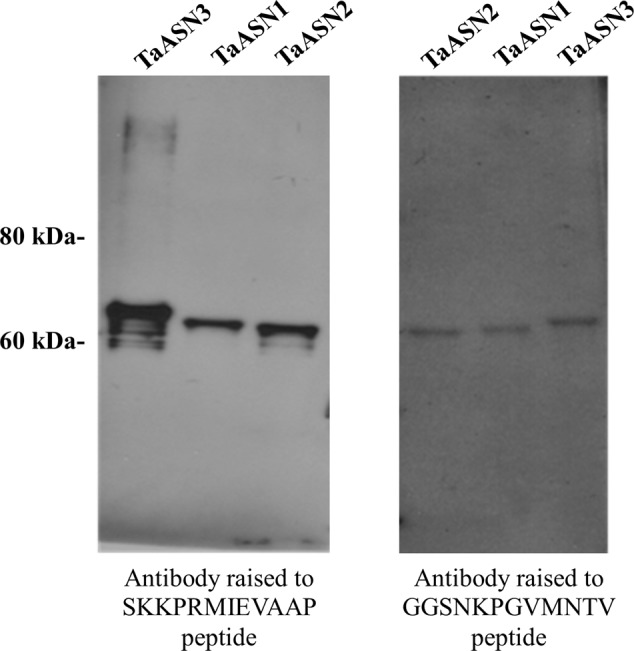
Western blot of heterologously expressed TaASN1, TaASN2, and TaASN3 proteins reacted with monoclonal antibodies raised to peptides SKKPRMIEVAAP and GGSNKPGVMNTV, as indicated.

A panel of monoclonal antibodies was raised to peptides (**Table 3**) corresponding to epitopes within the variable C-terminal regions of the proteins (**Figure 1**) with the aim of identifying antibodies that showed high specificity for the TaASN proteins and could distinguish between them. A western blot of the heterologously expressed TaASN1 proteins reacted with antibodies raised to two of the peptides, SKKPRMIEVAAP and GGSNKPGVMNTV, is shown in **Figure 4**. These two antibodies showed the highest specificity for the TaASN proteins, with the least non-specific binding, although all of the antibodies reacted with the TaASN proteins (not shown). However, none of the antibodies distinguished between the three asparagine synthetases. This was surprising because of the divergence of the amino acid sequences in this region (**Figure 1**). The SKKPRMIEVAAP epitope is present in TaASN1, while the GGSNKPGVMNTV epitope is at almost the same position in TaASN2 (indicated with a blue box in **Figure 1**). In each case there are only four identical residues and two conservative substitutions between the two proteins, and the similarity with TaASN3 is even lower. Nevertheless, TaASN1, TaASN2, and TaASN3 could be distinguished on the basis of size, with TaASN2 (65.06 kDa) migrating the furthest in the SDS-PAGE, followed by TaASN1 (65.49 kDa) and TaASN3 (66.24 kDa) (**Figure 4**).

**Table 3:** Epitopes used for the production of monoclonal antibodies for asparagine synthetases TaASN1, TaASN2, and TaASN3 from wheat.

Protein	Epitope identified from GenBank entries	Corresponding sequence in TaASN1–3 from cv. Spark	Position in protein
TaASN1 (GenBank AY621539)	HLPATIMAGTSK	HLPATILTGTSK	558–569
	IMAGTSKKPRMI	ILTGTSKKPRMI	563–574
	SKKPRMIEVAAP	SKKPRMIEVAAP	568–579
	MIEVAAPGVAIES	MIEVAAPGVAIES	573–585
TaASN2 (GenBank BT009049)	TVAVGGSNKPGV	TVAVGGSNKPGV	558–569
	GGSNKPGVMNTV	GGSNKPGVMNTV	562–573
	KPGVMNTVVPGV	KPGVMNTVVPGV	566–577
	MNTVVPGVAIET	MNTVVPGVAIET	570–581
TaASN3 (GenBank AK333183)	KAPASADPVFRP	KAPASVDPVLENAFHP	558–573
	DPVFRPPAHGES	DPVLENAFHPPAHGES	564–579
	PAHGESILVETG	PAHGESTLVKSA	574–585
	ILVETGVPAAAV	TLVKSAVPAAAV	580–591

*Underlined residues show where the sequences from cv. Spark differ from those in the GenBank entries.*

### Production of Asparagine and Glutamate in Reactions Catalyzed by Asparagine Synthetases, *TaASN1* and *TaASN2*

**FIGURE 5:**
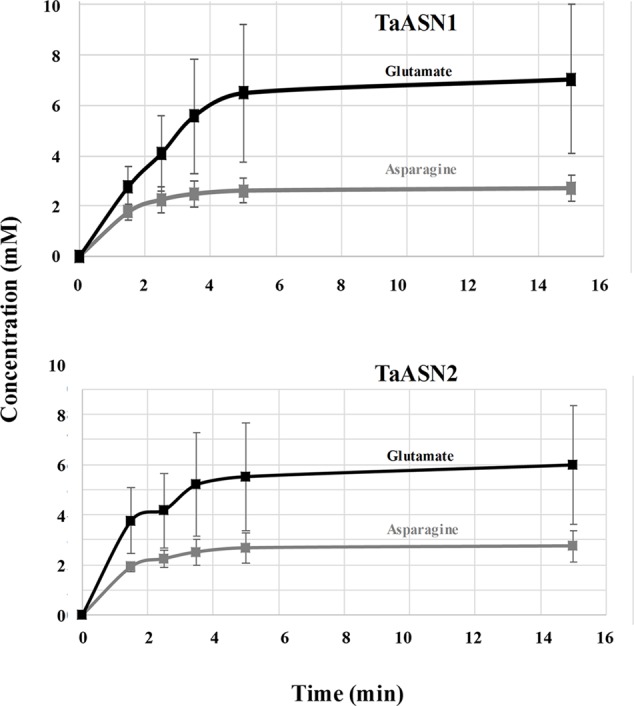
Plots (means with standard errors from two replicates) showing the synthesis of asparagine and glutamate, in reactions catalyzed by TaASN1 (top) and TaASN2 (bottom).

The production of asparagine and glutamate from aspartate and glutamine by TaASN1 and TaASN2 was measured in standard assays adapted from those described by Todd et al. (2008), Duff et al. (2011), and Kudiyarova et al. (2013). The reactions were sampled at 0, 1.5, 2.5, 3.5, 5, 15, 25, and 35 min and the asparagine and glutamate produced in the reaction were detected after conversion to a fluorescent derivative using *o*-phthalaldehyde reagent, separation by HPLC and detection of the fluorescent derivative with a scanning fluorescence detector. Both enzymes produced asparagine and glutamate, confirming that both were asparagine synthetases. The concentrations measured for the reactions are given in Supplementary File S1 for the two replicate assays done. Data from the calibration of the HPLC areas using standard concentrations of asparagine and glutamate are also given. The results up to the 15 min time point, by which time the concentrations of both asparagine and glutamate had plateaued, are shown graphically in **Figure 5**.

It was clear that the reactions catalyzed by both asparagine synthetases proceeded much more rapidly than had been reported for heterologously expressed maize or soybean enzymes (Todd et al., 2008; Duff et al., 2011). The reaction buffer contained 1.6 mM aspartate and 10 mM glutamine, meaning that glutamine was present in relative excess compared with aspartate. In both cases, the concentration of glutamate increased at a faster rate than the concentration of asparagine (**Figure 5**). By the 5-min time point in both reactions, the concentrations of both products were at or close to their maximum, indicating that the concentration of the reactants had become depleted. Notably, at this point, the concentration of glutamate was more than double that of asparagine, and the measured concentration of asparagine was actually slightly higher than would be expected given the starting concentration of aspartate.

### Modeling the Asparagine Synthetase Reaction

**FIGURE 6:**
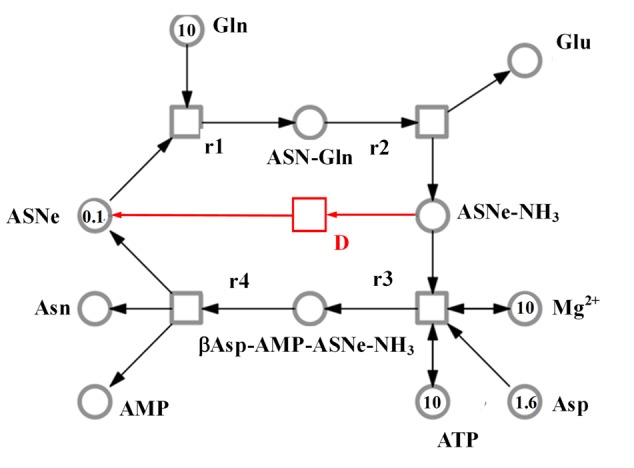
Model representing the reaction catalyzed by asparagine synthetase, comprising metabolites (circles), and reactions (squares). The concentration of metabolites is indicated abstractly by numbers in the circles. The model features 11 molecules: AMP, ATP, asparagine (Asn), glutamine (Gln), glutamate (Glu), aspartate (Asp), asparagine synthetase enzyme (ASNe), ASNe complexed with glutamine (ASNe–Gln), ASNe complexed with ammonia (ASNe–NH_3_), β-aspartyl-complex (βAsp–AMP–ASNe–NH_3_), and magnesium ions. Note that for clarity, water and pyrophosphate are not included. The model was generated assuming that the reactions follow mass action kinetics. The dissociation step for the ASNe–NH3 complex is highlighted in red.

The SNOOPY^®^ tool was used to construct a continuous Petri Net model which incorporates underlying ordinary differential equations (ODEs), representing the reaction catalyzed by asparagine synthetases, TaASN1 and TaASN2. The model was based on the reaction stages proposed by Gaufichon et al. (2010), with some modifications, and the experimental data, assuming mass action kinetics. A schematic diagram of the model is given in **Figure 6**. It comprises metabolites (“places” in Petri Net terminology) indicated by circles, and reactions (“transitions”) indicated by squares, connected by arrows (“edges”). The concentration of metabolites is represented abstractly by numbers on places. The model comprises one compartment (cell) with eleven molecules (species): adenosine monophosphate (AMP), asparagine (Asn), asparagine synthetase enzyme (for the purpose of the modeling annotated as ASNe), asparagine synthetase enzyme complexed with glutamine (ASNe-Gln), asparagine synthetase enzyme complexed with ammonia (ASNe–NH_3_), aspartate (Asp), adenosine triphosphate (ATP), β-aspartyl-complex (βAsp–AMP–ASNe–NH_3_), glutamine (Gln), glutamate (Glu) and magnesium ions (Mg^2+^). The four elementary biochemical reactions involved in the formation of asparagine are represented by the following equations:

• Reaction r1: ASNe + Gln → ASNe–Gln
• Reaction r2: ASNe–Gln → Glu + ASNe–NH_3_
• Reaction r3: ASNe–NH_3_ + Asp + ATP + Mg^2+^ (+ H_2_O) → βAsp–AMP–ASNe–NH_3_ + Mg^2+^ (+ PPi)
• Reaction r4: βAsp–AMP–ASNe–NH_3_ → Asn + ASNe + AMP

Note that H_2_O and PPi (pyrophosphate, P_2_O_7_^4-^) are in parentheses because they are ubiquitous and therefore were not included in the model. The behavior of each reaction is dependent on the corresponding parameter values and the initial concentrations of the metabolites. The model additionally includes a dissociation step for the ASNe–NH_3_ complex (reaction “D” highlighted in red in **Figure 6**), because this better fits the observed experimental data: Reaction D: ASNe–NH_3_ → ASNe

The following ODEs were generated by the SNOOPY^®^ Petri Net software (up to some naming adaptations to comply with the software requirements) to describe the mass action reactions determining the behavior of the 11 metabolites in the model:

(1) $$\begin{array}{c} d\;Gln/dt\;=\;-\;({k1}^{*}{\mathrm{Gln}}^{*}\mathrm{ASNe}) \end{array}$$

(2) $$\begin{array}{c} d\;ASNe-Gln/dt\;=\;({k1}^{*}{\mathrm{Gln}}^{*}\mathrm{ASNe})\;-({k2}^{*}ASNe-Gln) \end{array}$$

(3) $$\begin{array}{c} d\;ASNe/dt\;=\;({k4}^{*}\beta {Asp-AMP-ASNe-NH}_{3})\;+({\mathrm{kD}}^{*}{ASNe-NH}_{3})\;-\;({k1}^{*}{\mathrm{Gln}}^{*}\mathrm{ASNe}) \end{array}$$

(4) $$\begin{array}{c} dGlu/dt\;=\;({k2}^{*}ASNe-Gln) \end{array}$$

(5) $$\begin{array}{c} d\;{ASNe-NH}_{3}/dt\;=\;({k2}^{*}ASNe-Gln)\;-\;({k3}^{*}{ASNe- NH}_{3}^{*}\mathrm{Asp}{\;}^{*}{\mathrm{Mg}}^{2}{}^{+*}\mathrm{ATP})\;-\;({\mathrm{kD}}^{*}{ASNe-NH}_{3}) \end{array}$$

(6) $$\begin{array}{c} d\;Asp/dt\;=\;-\;({k3}^{*}{ASNe-NH}_{3}^{*}{\mathrm{Asp}}^{*}{\mathrm{Mg}}^{2+*}\mathrm{ATP}) \end{array}$$

(7) $$\begin{array}{c} d\;\beta {Asp-AMP-ASNe-NH}_{3}/dt\;=\;({k3}^{*}{ASNe-NH}_{3}^{*}\mathrm{Asp}\;{\;}^{*}\;{\mathrm{Mg}}^{2}{}^{+}\mathrm{ATP})\;-\;({k4}^{*}\beta {Asp-AMP-ASNe-NH}_{3}) \end{array}$$

(8) $$\begin{array}{c} d\;Asn/dt\;=\;({k4}^{*}\beta {Asp-AMP-ASNe-NH}_{3}) \end{array}$$

(9) $$\begin{array}{c} d\;AMP/dt\;=\;({k4}^{*}\beta {Asp-AMP-ASNe-NH}_{3}) \end{array}$$

(10) $$\begin{array}{c} d\;ATP/dt\;=\;0 \end{array}$$

(11) $$\begin{array}{c} d\;{\mathrm{Mg}}^{2}{}^{+}/dt\;=\;0 \end{array}$$

The model structure and corresponding induced behavior indicates that: (a) asparagine synthesis is dependent on the aspartic acid (aspartate), glutamine, and ATP concentration; (b) when aspartic acid is depleted but glutamine is still available, asparagine synthetase will continue to hydrolyze glutamine to glutamic acid, which is consistent with the observed experimental data (**Figure 5**); (c) if all substrates are available except for ATP (i.e., ATP would only be an input to the system, unlike in the current model), the limiting factor becomes ATP.

**FIGURE 7:**
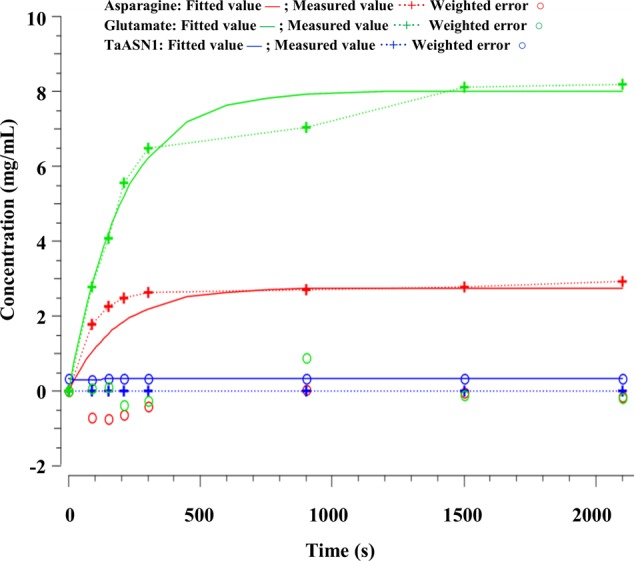
Time-series plots for parameter estimation against experimental data for TaASN1.

**FIGURE 8:**
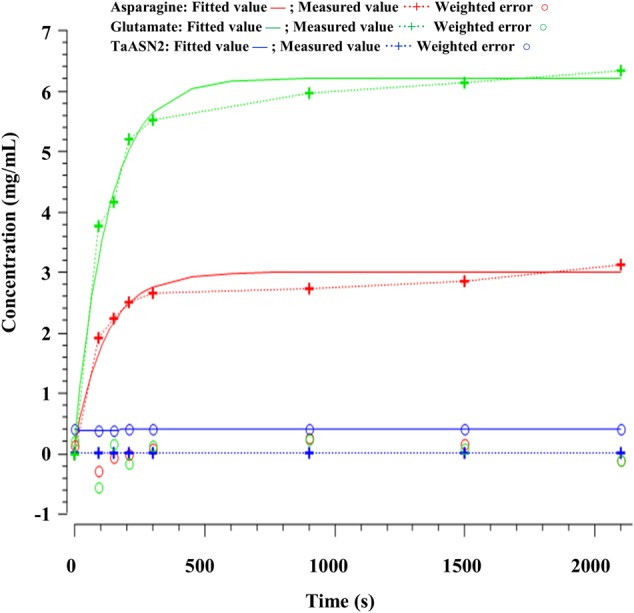
Time-series plots for parameter estimation against experimental data for TaASN2.

The parameters (**Table 4**) were determined using the parameter estimation function of Copasi (version 4.16; Hoops et al., 2006) based on the Hooke and Jeeves (1961) method. The values for the TaASN1 enzyme were defined using the following concentrations (mg/mL): ASNe between 1e - 06 and 1e + 06 with start value = 0.1; Glu between 1e - 06 and 1e + 06 with start value = 0.0; Asn between 1e - 06 and 1e + 06 with start value = 0.0, based on the data for Glu, Asn and ASNe provided in Supplementary File S1. The values for TaASN2 were the same, except for the start value of ASNe which was set at 0.09. The parameter estimation tasks were run for both enzymes for 2100 s with 2000 steps, size 1.05, with resulting rate values in units of mg/mL/s. The corresponding plots showing the time-series simulation results from the parameter fitting against the experimental data are given in **Figure 7** for TaASN1 and **Figure 8** for TaASN2, using initial concentrations of TaASN1 = 2.03 nmol/mL and TaASN2 = 2.10 nmol/mL.

**Table 4:** Rate parameters (mg/mL/s).

Rate parameter	TaASN1	TaASN2
k1	0.016	0.02
k2	3	3
k3	0.043	0.043
k4	10	10
kD	700	400

*Note that k1 refers to reaction r1 in the Petri Net model shown in **Figure 6**, and likewise the other parameters, while kD refers to the dissociation reaction D.*

## Discussion

Wheat is now known to contain four classes of asparagine synthetase genes, *TaASN1–4* (Gao et al., 2016). Our study established that there are single copies of *TaASN1, TaASN2*, and *TaASN4*, and two of *TaASN3*, and identified their chromosomal locations. The relatively simple structure of the gene family means that genetic interventions to reduce free asparagine accumulation and thereby acrylamide-forming potential in wheat grain are more likely to be successful. The antibodies raised in the study would be useful tools in the analysis of plants in which asparagine synthetase gene expression had been modified. The antibodies did not distinguish between TaASN1, TaASN2, and TaASN3, but it was possible to separate the enzymes on SDS-PAGE due to their slightly different sizes.

The study also showed that wheat asparagine synthetase enzymes, TaASN1 and TaASN2, can be expressed in *E. coli* and analyzed biochemically. Wheat asparagine synthetase activity has been measured before (Kudiyarova et al., 2013) but this was in plant extracts, so the results were not directly comparable to those obtained here. However, Todd et al. (2008) and Duff et al. (2011) analyzed heterologously expressed enzymes, the former from maize and the latter from maize and soybean. The reactions were not modeled, because their overall activity was very low, Todd et al. (2008) reporting a specific activity for asparagine production of 1–2 nmol/min/mg of protein. The reaction buffer used by Todd et al. (2008) contained 1.6 mM aspartate and 1 mM glutamine, while Duff et al. (2011) used a buffer containing 1.6 mM aspartate and 2 mM glutamine, and the reactions were sampled over a period of 60–90 min. In this study, a buffer was used containing 1.6 mM aspartate and 10 mM glutamine, as well as 10 mM ATP and 10 mM MgCl_2_, meaning that glutamine was present in relative excess compared with aspartate. The reactions catalyzed by the wheat asparagine synthetases proceeded much more rapidly than had been reported for the maize or soybean enzymes. By the 5-min time point, the concentrations of glutamate and asparagine were at or close to their maximum, indicating that the concentration of the reactants had become depleted.

A continuous Petri Net model based on mass-action kinetics was constructed using SNOOPY^®^ software to describe the reaction catalyzed by asparagine synthetase, and a set of differential equations was generated to describe each part of the reaction. It was notable from the experimental data that the concentration of glutamate increased at a faster rate than the concentration of asparagine. Indeed, the product concentrations for both enzymes plateaued with the concentration of glutamate more than double that of asparagine, although the ratio of glutamate to asparagine was higher for TaASN1 than TaASN2. This indicates that the early stages of the reaction (r1 and r2 in the model) can proceed faster than and independently of the later stages (r3 and r4), consistent with the hypothesis proposed by Gaufichon et al. (2010) that steps r1 to r4 occur sequentially rather than simultaneously. So, despite the overall equation of the reaction being Glutamine + Aspartate + ATP → Glutamate + Asparagine + AMP + PPi, glutamate synthesis can proceed independently of asparagine synthesis when aspartate is not available.

Modeling of the reactions catalyzed by TaASN1 and TaASN2 showed the two enzymes to be biochemically very similar except for the rate parameter (kD) for the dissociation step (**Table 4**). The careful fitting resulted in parameter values k1–k4 which were within expected biochemical ranges (Meister, 1974). The dissociation reaction, D, which we postulate in order to be able to fit the overall model to the data, is currently not described in the literature. However, the higher kD value for TaASN1 could explain the higher ratio of glutamate to asparagine produced in the TaASN1 reaction compared with the TaASN2 reaction.

Gene expression analyses have shown *TaASN1* and *TaASN2* to be the most highly expressed asparagine synthetase genes in wheat grain, with *TaASN2* expression rising to 10 times that of *TaASN1* by mid-development (Gao et al., 2016). Given this and the similarity in the biochemical data obtained for the two asparagine synthetases in the present study, bearing in mind that this was a single experiment with heterologously expressed enzymes, we conclude that TaASN2 is the major enzyme synthesizing asparagine in wheat grain, and therefore an appropriate target for genetic interventions to reduce free asparagine accumulation.

## Author Contributions

HX performed molecular cloning, expression, and biochemical analysis of wheat asparagine synthetases. TC performed modeling. SP performed statistical analyses. SR performed genomic analyses. RG performed nucleotide sequence analysis of wheat asparagine synthetases. JH is a Joint project leader. MH and DG performed modeling. NH is a Joint project leader and lead author.

## Conflict of Interest Statement

The project was supported by a consortium of companies and organizations from the wheat supply chain, comprising PepsiCo, Nestlé, Cereal Partners UK, Weetabix, Kelloggs, United Biscuits, Con Agra Foods, Lantmännen, Saaten Union, the Agriculture and Horticulture Development Board, CEEREAL, the Association of Cereal Food Manufacturers and SNACMA. This consortium did not participate in the experimental design or execution, or in the presentation or interpretation of the results in this report.

## Footnotes

1 http://www.geneious.com

2 http://plants.ensembl.org/Triticum_aestivum/Tools/Blast?db=core;tl=Igru1o47ao3Q1dXo-13756266

3 http://www-dssz.informatik.tu-cottbus.de/DSSZ/Software/

4 http://www.brenda-enzymes.org/

5 www.cerealsdb.uk.net
